# Reduction in Obesity-Related Hepatic Fibrosis by SR1664

**DOI:** 10.3390/biology12101287

**Published:** 2023-09-26

**Authors:** Benita L. McVicker, Ronda L. Simpson, Frederick G. Hamel, Robert G. Bennett

**Affiliations:** 1 Research Service, Nebraska-Western Iowa Health Care System, Omaha, NE 68105, USA; bmcvicker@unmc.edu (B.L.M.); frederick.hamel@va.gov (F.G.H.); 2 Departments of Internal Medicine and Pharmacology & Experimental Neuroscience, University of Nebraska Medical Center, Omaha, NE 68198, USA; 3 Departments of Internal Medicine and Biochemistry & Molecular Biology, University of Nebraska Medical Center, Omaha, NE 68198, USA

**Keywords:** hepatic fibrosis, peroxisome-proliferator-activated receptor gamma, cirrhosis

## Abstract

**Simple Summary:**

Obesity can contribute to the development of liver disease, including the accumulation of excess collagen (fibrosis), which leads to organ dysfunction. Drugs that target peroxisome-proliferator-activated receptor gamma (PPARγ), a protein that controls genes involved in fat storage and fibrosis, have been used to treat diabetes, but can cause unwanted side-effects. Newer drugs have been designed that selectively target PPARγ to retain the antidiabetic effects, but without the side-effects such as weight gain and bone loss, but less is known about whether they reduce fibrosis. We hypothesized that one of these new drugs (SR1664) would improve the liver fibrosis associated with obesity. We used mice fed a diet high in fat and carbohydrates to induce fatty liver with fibrosis, and then determined the effect of SR1664. We found that SR1664 did not improve insulin sensitivity, but effectively reduced liver fibrosis, without causing weight gain. These findings support the use of selective PPARγ drugs for the treatment of liver fibrosis.

**Abstract:**

Peroxisome-proliferator-activated receptor gamma (PPARγ) is a transcription factor with adipogenic, insulin-sensitizing, and antifibrotic properties. Strong PPARγ activators, such as the thiazolidinediones, can induce unwanted effects such as edema, weight gain, and bone loss, and therefore selective modulators of PPARγ are in development. We previously reported that one selective PPARγ modulator, SR1664, reduced toxin-induced hepatic fibrosis and the activation of hepatic stellate cells (HSCs), the main collagen-producing liver cell in fibrosis. In this study, we used a high fat and high carbohydrate (HFHC) model of hepatic steatosis and fibrosis to determine the effect of SR1664. Mice were placed on a standard chow or HFHC diet for 16 weeks, with SR1664 or control treatment for the final 4 weeks. SR1664 did not alter weight gain or fasting insulin or glucose levels. The size of lipid droplets in the HFHC group was reduced by SR1664, but there was no effect on total liver triglyceride levels. The degree of fibrosis was significantly reduced by SR1664 in mice on the HFHC diet, and this was accompanied by a decrease in activated HSC. In summary, SR1664 improved insulin sensitivity and reduced fibrosis in the HFHC diet, suggesting selective PPARγ modulation is effective in obesity-related liver fibrosis.

## 1. Introduction

Peroxisome-proliferator-activated receptor gamma (PPARγ) is a transcription factor best known for its regulation of adipogenesis and insulin-sensitizing properties in a number of tissues [1]. The latter property led to the development of PPARγ agonists as diabetes medications. In particular, the thazolidinedione (TZD) drugs, such as rosiglitazone and pioglitazone, are strong PPARγ agonists, and became widely clinically used for diabetes treatment. There is also evidence that TZDs can reduce tissue fibrosis in a number of organs [2]. However, the TZD drugs have some side-effects, including weight gain, increased adipose tissue mass, edema, and bone loss [1]. Therefore, alternative PPARγ activators are being developed to minimize these negative effects.

Recent findings into the mechanism of action of TZDs and other PPARγ activators have led to the development of selective modulators of PPARγ activity. One finding was the discovery of an inhibitory phosphorylation of PPARγ by cyclin-dependent kinase-5 (CDK5), and that blocking this phosphorylation promotes insulin sensitization but not the adipogenic program [3]. The TZD drugs not only block this phosphorylation but also promote a strong adipogenic program with adverse effects that include edema, weight gain, and risk of cancer, which have led to their prohibition in many countries [1]. Newer selective PPARγ modulators have insulin-sensitizing properties, without promoting adipogenesis and other adverse effects. One of these, SR1664, blocks CDK5-mediated pSer273 equally as well as TZDs, improves insulin sensitivity and glucose tolerance, but does not promote adipogenesis, weight gain, edema, or bone loss [3,4].

In the liver, PPARγ regulates the activation state of the hepatic stellate cells (HSCs) [1]. The HSC in the normal, quiescent state serves as retinoid-storing cells. When activated in response to tissue injury or insult, it transdifferentiates to an activated, myofibroblastic phenotype, responsible for the excess collagen accumulation that leads to fibrosis. PPARγ expression is high in quiescent HSC but is reduced with HSC activation [5]. Increased PPARγ expression or activity induced a reversion of HSC to the quiescent phenotype, with decreased collagen expression [6]. TZDs prevented hepatic fibrosis in vivo [7,8,9], leading to interest in TZDs for hepatic fibrosis treatment. However, further studies suggested that TZDs were ineffective for established liver fibrosis in rodents, casting doubt on their utility for this purpose [10,11]. For this reason, we began to study whether SR1664 retained the antifibrotic effects of the TZD drugs. We found that SR1664 reduced fibrotic markers in HSC in vitro and reduced hepatic fibrosis in the mouse carbon tetrachloride model in vivo [12]. In the present study, we sought to use a diet-induced obesity-induced model of hepatic fibrosis with insulin resistance to determine if SR1664 would reduce collagen accumulation in this model.

## 2. Materials and Methods

Animals: All animal studies were performed in accordance with The Guide to the Care and Use of Laboratory Animals (8th edition, 2011) and were approved by the VA-NWI Institutional Animal Care and Use Committee. Male C57BL6/J mice (Jackson Laboratories) aged 7 weeks were randomly assigned (12 per group) to receive either regular chow diet or a high-fat + high-carbohydrate (HFHC) diet consisting of 58% calories from fat (Surwit diet, Research Diets, New Brunswick, NJ, USA) with the drinking water supplemented with 42 g/L of carbohydrates (45% sucrose, 55% fructose) as described [13]. The diet was administered for 16 weeks. During the final 4 weeks, 6 mice from each group were randomly assigned to receive 20 mg/kg/day of SR1664 (Cayman Chemical, Ann Arbor, MI, USA) delivered orally in a 100 µL peanut butter pellet, or peanut butter pellets alone as described previously [12] for a further 4 weeks. Two days before the end of treatment, the total body fat and lean mass were determined using an Echo-MRI 3-In-1 instrument.

Serum and tissue measurements: Serum was collected and analyzed for alanine transaminase (ALT) and aspartate transaminase (AST) using automated analysis in the VA-NWI Clinical Chemistry Laboratory. Fasting (6 h fast with carbohydrate-free drinking water) serum insulin levels were measured using ELISA (Mercodia, Uppsala, Sweden) according to the manufacturer’s instructions. Fasting glucose levels were measured using a glucometer (Accuchek). Insulin resistance index (HOMA-IR) was calculated as the fasting glucose (mg/dL) × fasting insulin (nmol/L)/22.5. The liver triglyceride level was determined using the Triglyceride Quantification kit (Sigma-Aldrich, St. Louis, MO, USA).

Histology: Formalin-fixed paraffin-embedded sections were prepared and stained for hematoxylin and eosin by the Tissue Sciences Facility at the University of Nebraska Medical Center. Total collagen content was determined through Sirius Red staining as described previously [14]. Five random non-overlapping images were collected from each specimen. The staining was quantified using ImageJ software (version 1.52a) using automated color deconvolution and threshold functions, and is expressed as % Sirius Red-positive area as a function of the total tissue area.

Immunohistochemistry: Formalin-fixed paraffin-embedded sections were dewaxed through graded ethanol washes, and then subject to heat-induced epitope retrieval by heating in Tris-EDTA pH 9.0 in a pressure cooker. Endogenous peroxidase was blocked with Bloxall (Vector Laboratories, Newark, CA, USA) for 10 min, and then a protein block (50 mM Tris pH 7.4 containing 5% normal goat serum) was applied for 30 min. Rabbit anti-α-smooth muscle actin (SMA) antibody (#19245, Cell Signaling Technology, Danvers, MA, USA, 1:2000) or rabbit anti-collagen-1 antibody (#72026, Cell Signaling Technology, 1:200) was applied overnight at 4 °C. The secondary antibody (SignalStain Boost goat anti-rabbit HRP, #8114, Cell Signaling Technology) was applied for 30 min at room temperature, and the detection was carried out using tyramide signal amplification using AlexaFluor-647-labeled tyramide (ThermoFisher, Lafayette, CO, USA) as described [15]. After counterstaining with DAPI, autofluorescence was quenched with 0.1% Sudan Black B in 70% ethanol for 20 min, and then slides were mounted using Prolong Gold (ThermoFisher). Five random non-overlapping images were captured using a 20X objective, and the SMA staining was calculated using ImageJ software using the triangle threshold algorithm.

Gene expression assays: Total RNA was extracted from liver tissue using Trizol reagent followed by the Purelink RNA Miniprep kit (ThermoFisher) with on-column DNAse treatment as per the manufacturer’s instructions. The RNA was quantified using the Ribogreen assay, and RNA integrity was verified by visualization on agarose gels. RNA (1 µg) was converted to cDNA using the High-Capacity cDNA Reverse Transcription kit (Applied Biosystems, Lafayette, CO, USA). Gene expression was determined using probe-based assays (see Supplemental Materials) containing 1 µL of cDNA in a total reaction volume of 20 µL using iTaq Master Mix (Bio-Rad Laboratories, Hercules, CA, USA) and a CFX Connect real-time PCR system (Bio-Rad). The expression of each gene was calculated relative to that of two housekeeping genes (*Tbp* (TATA binding protein) and *B2m* (β-2 microgloblin)) using the 2^ΔΔCt^ method, and normalized to the expression level of the chow-fed group.

Matrix metalloproteinase (MMP) activity assays—Liver homogenates were prepared in Tris (tris(hydroxymethyl)aminomethane) buffer pH 7.6 containing 0.1% Triton X-100 using a FastPrep 24 instrument (MP Biomedicals, Irvine, CA, USA). The homogenates were cleared through centrifugation at 10,000× *g* for 10 min at 4 °C, and then protein content was determined using the BCA (bicinchoninic acid) assay (ThermoFisher). A total of 20 µg of homogenate protein was used to determine enzyme activity using the SensoLyte 520 MMP3 or MMP13 assay kits (AnaSpec, Fremont, CA, USA) according to the manufacturer’s instructions. The 60 min endpoint protocol was used, and the activity was determined by reading fluorescence (excitation 490 nm, emission 520 nm) in a SpectraMax M5 plate reader (Molecular Devices, San Jose, CA, USA).

Statistical analysis: All statistical analyses were performed using GraphPad Prism 9 software. All data were analyzed for normal distribution using the Shapiro–Wilk test. Parametric data were analyzed for statistical significance using one-way or two-way analysis of variance as appropriate, with Tukey’s post test. Non-parametric data were analyzed using one-way or two-way analysis of variance as appropriate, with the Kruskal–Wallis post test. A value of *p* < 0.05 was defined as significant.

## 3. Results

**Figure 1:**
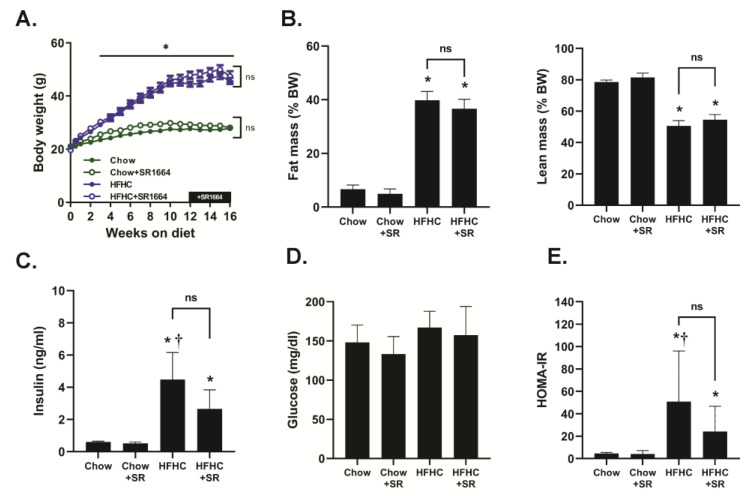
Effect of HFHC feeding and SR1664 treatment on body and serum parameters. (**A**) Body weights were measured weekly throughout this study. * *p* < 0.05 through 2-way ANOVA, *n* = 6, ns: not significant. (**B**) Body mass composition at the end of this study. Data are expressed as the fat or lean mass as a percent of total body mass * *p* < 0.05 vs. chow or chow + SR by ANOVA, *n* = 6, ns: not significant. (**C**–**E**) Fasting insulin, glucose, and HOMA-IR were determined at the end of this study. * *p* < 0.05 vs. chow + SR, † *p* < 0.05 vs. chow by ANOVA, *n* = 6, ns: not significant.

Mice receiving the HFHC diet steadily gained weight throughout this study, and they had a significantly increased body weight compared to chow-fed mice beginning at 3 weeks of feeding (Figure 1A). There was no significant effect of SR1664 treatment on the body weights of either chow-fed or HFHC-fed mice. The HFHC diet caused a significant increase in % fat mass and a decrease in % lean mass compared to chow-fed mice, but SR1664 had no significant effect on body mass composition with either diet (Figure 1B). Similarly, fasting insulin levels were elevated with the HFHC diet compared to chow-fed mice with or without SR1664 treatment (Figure 1C). Treatment with SR1664 did not significantly reduce serum insulin levels in either group. Fasting glucose levels were not significantly different between any of the groups (Figure 1D). The HOMA-IR index suggested that the HFHC caused insulin resistance, but that SR1664 did not significantly improve insulin resistance (Figure 1E).

**Figure 2:**
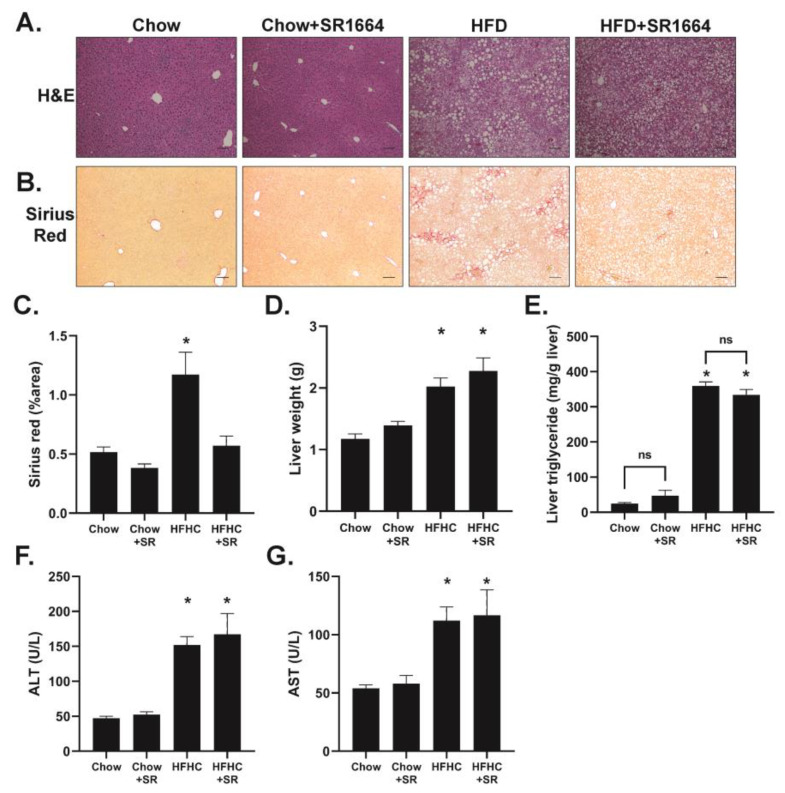
Effect of HFHC feeding and SR1664 on liver parameters. (**A**,**B**) Hematoxylin and eosin, and Sirius red staining of liver tissue, respectively. Scale bar = 100 μm. (**C**) Quantification of Sirius red staining. (**D**) Total liver weight. (**E**) Total hepatic triglyceride content. (**F**,**G**) Serum ALT and AST levels. For all data, * *p* < 0.05 vs. all other groups through ANOVA, *n* = 6. ns: not significant.

The livers from HFHC-diet-fed mice had a significantly increased lipid content as visualized through hematoxylin and eosin staining (Figure 2A). The SR1664 treatment had no obvious effect on the liver in mice on the chow diet, but the lipid droplet size in the HFHC diet appeared to be smaller, with fewer large lipid droplets overall. Both the liver weight and liver tissue triglyceride content was elevated in both HFHC groups and was not significantly decreased through SR1664 treatment (Figure 2D,E). These data suggest that SR1664 may have affected lipid droplet size but did not significantly reduce the total liver triglyceride content. While the HFHC diet increased total liver weight and serum levels of ALT and AST, ST1664 had no significant effect on these parameters (Figure 2F,G).

Total collagen content was visualized through Sirius red staining of liver tissue (Figure 2B). The staining in the chow-diet-fed livers was limited to the large perivenular areas and was not affected by SR1664. The HFHC diet caused increased collagen content in the liver parenchyma, particularly in areas with large lipid droplets. The degree of parenchymal collagen deposition was markedly less in the HFHC livers after SR1664 treatment. Quantification of the Sirius red staining confirmed a significant decrease in liver collagen content with SR1664 treatment (Figure 2C).

**Figure 3:**
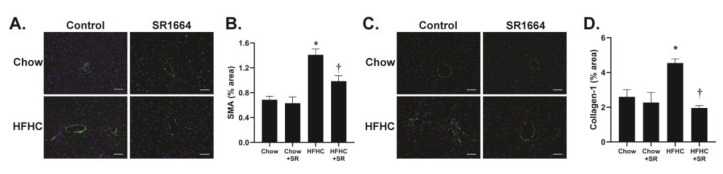
SMA and collagen-1 content in liver tissue. (**A**) Fluorescent images of SMA (green) counterstained with DAPI (blue) to visualize nuclei. Scale bar: 50 μm. (**B**) Quantification of SMA staining. * *p* < 0.05 vs. all other groups, † *p* < 0.05 vs. HFHC through ANOVA, *n* = 6. (**C**) Fluorescent images of collagen-1 (green) counterstained with DAPI (blue) to visualize nuclei. Scale bar: 50 μm. (**D**) Quantification of collagen-1 staining. * *p* < 0.05 vs. all other groups, † *p* < 0.05 vs. HFHC through ANOVA, *n* = 3.

The main cells responsible for collagen production in liver fibrosis are the hepatic stellate cells (HSCs). The expression of SMA is a marker of activated (profibrotic) HSC. Immunohistochemical analysis of liver tissue revealed that in the chow-fed mice, SMA expression was limited to areas around large veins (Figure 3A). With HFHC feeding, the SMA expression was seen extending into the parenchyma, particularly in areas populated with large lipid droplets, similar to the Sirius red staining. SR1664 treatment significantly decreased SMA content, suggesting decreased activation of HSC (Figure 3B). Similarly, the presence of collagen type 1 was limited to perivenular areas in the chow-fed groups, but had infiltrated the parenchymal region after HFHC diet feeding (Figure 3C). Treatment with SR1664 significantly decreased collagen-1 content (Figure 3D). Therefore, the data suggest that in diet-induced hepatic steatosis with fibrosis, SR1664 reduces total and type 1 collagen and HSC activation.

**Figure 4:**
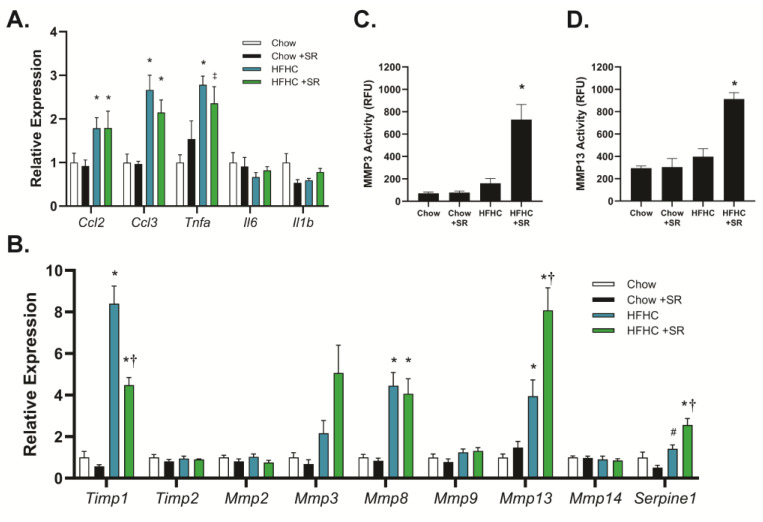
Expression and activity of inflammation and MMP-related genes. (**A**,**B**) Expression levels of genes related to inflammation (**A**) or fibrosis (**B**). * *p* < 0.05 vs. chow or chow + SR groups, ‡ *p* < 0.05 vs. chow, # *p* < 0.05 vs. chow + SR, † *p* < 0.05 vs. HFHC through ANOVA, *n* = 4–6. (**C**,**D**) Enzymatic activity of MMP3 (**C**) or MMP13 (**D**) in liver homogenates. * *p* < 0.05 vs. all other groups through ANOVA, *n* = 3.

The role of SR1664 in the expression of genes related to inflammation and fibrosis was determined using quantitative PCR to determine potential mechanisms of action. The HFHC diet increased expression of the inflammation-related genes chemokine (C-C) motif ligand-2 (*Ccl2*, also known as macrophage inflammatory protein-1α), chemokine (C-C) motif ligand-3 (*Ccl3*, also known as monocyte chemoattractant protein-1), and tumor necrosis factor-α (*Tnfα*), but not interleukin-1b (*Il1b*) or interleukin-6 (*Il6*) (Figure 4A). Treatment with SR1664 did not significantly affect the expression of any of these genes with either diet, suggesting that reduced inflammation was not a major factor in the decreased fibrosis. In contrast, the HFHC diet markedly increased the expression of tissue inhibitor of metalloproteinases-1 (*Timp1*), and SR1664 treatment significantly reduced *Timp1* expression (Figure 4B). *Timp2* was not affected by diet or SR1664 treatment. In addition, SR1664 significantly increased the expression of the matrix metalloproteinases *Mmp3* and *Mmp13* compared to the HFHC diet, but not *Mmp2*, *Mmp8, Mmp9*, or *Mmp14*. Interestingly, SR1664 also significantly increased plasminogen activator inhibitor-1 (*Serpine-1*) expression.

To verify that the combination of increased *Mmp* expression and decreased *Timp*1 expression resulted in altered MMP activity, liver homogenates were assessed for MMP3 and MMP13 activity using fluorescent-substrate-based assays. Treatment with SR1664 significantly increased MMP3 activity in the HFHC model compared to animals on the HFHC diet alone (Figure 4C). A similar pattern was seen with MMP13 activity (Figure 4D). Therefore, the mechanism for the antifibrotic effect of SR1664 may be increased extracellular matrix degradation due to increased MMP expression coupled with reduced TIMP1 expression, resulting in elevated MMP3 and MMP13 activity.

## 4. Discussion

Despite early studies showing that the full PPARγ thiazolidinedione agonists could inhibit HSC activation in vitro [7,16,17] and prevent hepatic fibrosis in some rodent models [7,8,9,18,19], studies of their effects in the more clinically relevant models of established hepatic fibrosis have yielded conflicting results. Some studies using cholestatic or toxin-induced or diet-induced hepatic fibrosis models found no reduction in hepatic fibrosis with thiazolidinediones [10,11,20,21]. Other studies, including high-fat–high-cholesterol or choline-methionine-deficient diet models, found a reduction in fibrosis [22,23,24]. The reasons for the discrepancy in these results are still not definitively known but may relate to the strong adipogenic transcriptional program triggered by these agents, to well-known side-effects such as weight and adipose tissue gain and edema, or to the insulin resistance state induced by the model.

As mentioned earlier, full agonists of PPARγ activate a strong adipogenic transcriptional program that can result in weight gain, increased adipose tissue mass, edema, and bone loss [25]. For this reason, much attention has been recently given to the development of selective PPARγ modulators (SPPARγMs), which are selective, partial, or “non-agonist” PPARγ modulators. Many of these agents, including SR1664, act by blocking an inhibitory phosphorylation of PPARγ by the cyclin-dependent kinase 5 (CDK5) [3,4]. This allows a basal transcriptional activity of PPARγ that accounts for its insulin-sensitizing properties without the adipogenic program. Indeed, SR1664 was shown to improve insulin sensitivity in a high-fat-diet-feeding model, but its effects on models of fibrosis were unclear. Our previous study showed that SR1664 improved established carbon-tetrachloride-induced hepatic fibrosis, suggesting that the antifibrotic effects of PPARγ may be mediated through its non-adipogenic transcriptional activity [12]. In this study, we sought to determine the effect of SR1664 in a diet-induced model of obesity, steatosis, and hepatic fibrosis.

Previous studies using selective PPARγ activators, including SR1664, suggested that these agents are effective in improving insulin resistance through their insulin-sensitizing effects [4]. In the present study, we observed insulin resistance (through elevated fasting insulin levels) and increased liver triglyceride content in response to the HFHC diet treatment. However, although there was a trend toward reduction, SR1664 treatment failed to significantly improve either of these parameters. The reason for this is unclear, but may be related to differences in the diet, in that previous studies did not supplement the drinking water with carbohydrates. Another possibility is that the oral delivery of SR1664 did not produce sufficient bioavailability, as its oral pharmacokinetics are not optimal [26]. Although our previous study showed that oral SR1664 treatment could result in physiological effects, these were not tested in diet-induced obesity models [12]. It is also possible that, in the presence of continued administration of the HFHC diet, a longer SR1664 treatment may be necessary. The development of new drugs based on SR1664 with improved oral pharmacokinetics may provide better effects in obese models [26,27].

Despite the failure of SR1664 to significantly improve insulin sensitivity, it significantly reduced liver collagen content. This was accompanied by a reduction in the number of activated HSCs as determined by the presence of SMA expression. These results were similar to our previous study showing that SR1664 significantly reduced toxin-induced liver injury [12]. It is interesting that despite the lack of effectiveness of SR1664 on insulin resistance, the antifibrotic effect was quite effective, suggesting that the HSC was highly sensitive to its effects.

Hepatic fibrosis results from multiple processes, with inflammation as a critical early step [28]. Activation of Kupffer cells, the resident hepatic macrophages, as well as infiltrating inflammatory cells, results in the release of cytokines that contribute to HSC activation. The HFHC diet resulted in increased expression of three inflammatory cytokines, *Ccl2, Ccl3,* and *Tnfa*, while there was no significant change in *Il1b* or *Il6* expression. There was no effect of SR1664 on the expression of any of these genes, either with the chow or HFHC diet. These results suggest that SR1664 does not reduce diet-induced hepatic fibrosis via modulation of inflammation.

The levels of collagens and other extracellular matrix proteins are regulated by a balance between protein production and degradation. The enzymes most responsible for extracellular matrix degradation are the MMPs [29]. The activity of MMPs is regulated by their endogenous inhibitors, the TIMPs. The balance between the levels of MMPs and TIMPs is an important factor in the regulation of matrix protein abundance. In this study, we observed a significant increase in *Timp1* expression with the HFHC diet, which was significantly decreased by SR1664. At the same time, SR1664 increased both the expression and activity of *Mmp3* and *Mmp13*. Importantly, MMP13 is a major enzyme responsible for the degradation of fibrillary collagen, such as collagen type 1. In addition to matrix degradation, an important role of MMP3 is in the activation of other MMPs from their proenzyme forms. The finding of an SR1664-mediated elevation in the gene encoding PAI-1 (*Serpine1*) was somewhat unexpected, as increased PAI-1 levels are associated with advanced hepatic fibrosis [30]. However, one study showed that the PPARγ agonist rosiglitazone increased *Serpine1* expression in a diet-induced obesity model, and therefore the role of PAI-1 is unclear [31]. Together, the data suggest that SR1664 alters the MMP/TIMP1 balance to favor matrix degradation.

There were differences in the results observed in the present study and the original publication describing the HFHC diet [13]. While the changes in body weight, body mass composition, and fasting insulin levels were comparable, we did not observe a difference in fasting glucose with the HFHC diet. The reason for this is unclear but may be due to differences in the fasting duration. The previous study used an overnight fasting period, while we used a 6 h fast to avoid a starvation response and depletion of liver glycogen stores. In addition, during the fasting period, we also removed the sucrose and fructose from the drinking water to minimize any effects of these carbohydrates on the glucose measurements, but it is not clear if this was performed in the previous study.

## 5. Conclusions

In summary, SR1664 treatment reduced liver fibrosis in a mouse model of high-fat- and high-carbohydrate-diet-induced obesity. The antifibrotic effect occurred even in the absence of an improvement in insulin resistance in the model. This raises the possibility that the antifibrotic effect may occur through a different mechanism or target gene set than the insulin-sensitizing effects, and underscores the need to conduct further studies with the next-generation drugs in this class.

## Data Availability

The raw data will be made available upon request.

## Supplementary Materials

The following supporting information can be downloaded at https://www.mdpi.com/article/10.3390/biology12101287/s1, Table S1: Gene expression assays.
