# Supplementary material for: Reduction in Obesity-Related Hepatic Fibrosis by SR1664
Source: Biology (Basel). 2023 Sep 26;12(10):1287. doi: 10.3390/biology12101287 (PMC10604321; doi:10.3390/biology12101287)
Supplement: Supplementary file 1 [file biology-12-01287-s001.zip › biology-2596678-supplementary.pdf]

Supplemental Table S1: Gene expression assays

**Gene name**	**Gene symbol**	**Assay number**	**Probe context sequence**
Beta-2 microglobulin	*B2m*	Mm00437762_m1	tctcactgaccggcctgtatgctat
Chemokine (C-C) motif ligand 2	*Ccl2*	Mm00441242_m1	ctcagccagatgcagttaacgcccca
Chemokine (C-C) motif ligand 3	*Ccl3*	Mm00441258_m1	gtcttctcagcgccatatggagctg
Interleukin-1b	*Il1b*	Mm00434228_m1	gaccccaaaagatgaagggctgctt
Interleukin-6	*Il6*	Mm00446190_m1	tgagaaaagagttgtgcaatggcaa
Matrix metalloproteinase-2	*Mmp2*	Mm00439506_m1	ggctgtgttcttcgcagggaatgag
Matrix metalloproteinase-3	*Mmp3*	Mm00440295_m1	gatgtcactggtaccaacctattcc
Matrix metalloproteinase-8	*Mmp8*	Mm00439509_m1	ctgagaattacctacgaaaattct
Matrix metalloproteinase-9	*Mmp9*	Mm00442991_m1	tccagtaccaagacaaagcctatt
Matrix metalloproteinase-13	*Mmp13*	Mm00439491_m1	gcagttccaaaggctacaacttgtt
Matrix metalloproteinase-14	*Mmp14*	Mm00485054_m1	ccccgaagcctggctgcagcagtat
Plasminogen-activator inhibitor-1	*Serpine1*	Mm00435858_m1	tcatcctgcctaagttctctctgga
TATA-box binding protein	*Tbp*	Mm01277045_m1	ttaatctacagaatgatcaaacccag
Tissue inhibitor of metalloproteinases-1	*Timp1*	Mm01341361_m1	ggctgtggggtgtgcacagtgtttc
Tissue inhibitor of metalloproteinases-2	*Timp2*	Mm00441825_m1	tgcaagatcactcgctgtcccatga
Tumor necrosis factor-α	*Tnfa*	Mm00443258_m1	cccaaagggatgagaagttcccaaa
